# miRNA-dysregulation associated with tenderness variation induced by acute stress in Angus cattle

**DOI:** 10.1186/2049-1891-3-12

**Published:** 2012-06-01

**Authors:** Chunping Zhao, Fei Tian, Ying Yu, George Liu, Linsen Zan, M Scott Updike, Jiuzhou Song

**Affiliations:** 1 College of Animal Science and Technology, Northwest A&F University, Yangling, Shaanxi, 712100, China; 2 Department of Animal & Avian Sciences, University of Maryland, College Park, MD, 20742, USA; 3 Standards Division, USDA-Agricultural Marketing Service - National Organic Program, Washington D.C., 20250, USA; 4 Department of Animal Breeding and Genetics, College of Animal Sciences, China Agricultural University, Beijing, 100193, China; 5 Bovine Functional Genomic Laboratory, Animal and Natural Resources Institute, USDA-Agricultural Research Service, Beltsville, MD, 20705, USA

**Keywords:** miRNA, Bovine, Beef tenderness, Stress

## Abstract

miRNAs are a class of small, single-stranded, non-coding RNAs that perform post-transcriptional repression of target genes by binding to 3’ untranslated regions. Research has found that miRNAs involved in the regulation of many metabolic processes. Here we uncovered that the beef quality of Angus cattle sharply diversified after acute stress. By performing miRNA microarray analysis, 13 miRNAs were significantly differentially expressed in stressed group compared to control group. Using a bioinformatics method, 135 protein-coding genes were predicted as the targets of significant differentially expressed miRNAs. Gene Ontology (GO) term and Ingenuity Pathway Analysis (IPA) mined that these target genes involved in some important pathways, which may have impact on meat quality and beef tenderness.

## Introduction

MicroRNAs are one of the largest gene families and account for ~1% of the genome [1]. They are 21–25 nucleotide small, non-coding RNAs that post-transcriptionally repress the expression of protein-coding genes through binding to the 3’ untranslated regions (UTR) of the target mRNAs [1-5]. Accumulated evidence indicates that miRNAs are important in the regulation of many biological processes, such as developmental timing, cell metabolism, cell differentiation, cell death, cell proliferation, haematopoiesis and patterning of the nervous system, *etc*[1,4,6]. Recent studies have uncovered muscle-specific miRNAs that regulate diverse aspects of muscle function, including myoblast proliferation, differentiation, contractility and stress responsiveness [7-10]. Disruption of miRNA biogenesis causes diverse developmental defects, including abnormal embryogenesis and depletion of stem cells [4]. It has been reported that microRNA-133a regulates cardiomyocyte proliferation and suppresses smooth muscle gene expression in heart [8]. miR-1 and miR-133 have distinct roles in modulating skeletal muscle proliferation and differentiation in cultured myoblast *in vitro* and in Xenopus laevis embryos *in vivo*[9]. miR-335 and miR-126 are identified as metastasis suppressors in human breast cancer because their expressions are lost in the majority of primary breast tumors [11]. Additionally, miRNAs have been found involved in viral infections, cancer, cardiovascular disease and neurological and muscular disorders [6,12-20]. With the progression of research, a large number of miRNAs have been found to play roles in the regulation of metabolic process. Although there are 18226 entries in miRBase, representing hairpin precursor miRNAs and expressing 21643 mature miRNA products in 168 species, only a handful of miRNAs have been studies deeply, and a range of functions extending beyond developmental regulation have been revealed [4]. Especially, 665 miRNAs in bovine are shown in the database, and some of them are studied in bovine cell *in vitro*, but few have been studied *in vivo*[21,22].

Beef tenderness is a complex characteristic influenced by many aspects, such as production, processing factors and cooking aspects, *etc*. More efforts have been focused on factors influencing meat quality, including breed, sex, feed, handling, environment, finishing weight and age at slaughter, *etc*[23-28]. So far, no research is performed on whether the variation of beef tenderness is regulated by miRNAs. To test our hypothesis that acute stress may influence beef quality mediated by miRNAs, a miRNA microarray was used to detect differentially expressed miRNAs between stressed and non-stressed groups of cattle. The results from the study demonstrated that acute stress altered both beef quality and miRNA expression, which will help us identify mechanisms underlying the control of beef tenderness.

## Results

### Differentially expressed miRNAs in LD muscle with differential stress status

**Figure 1:**
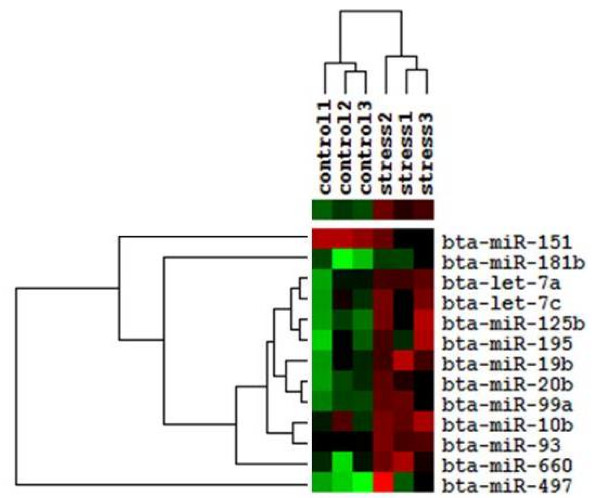
**Cluster analysis of significant differentially expressed miRNAs in microarray.** These miRNAs were visualized with Treeview after hierarchical clustering. Each miRNA is represented by a single row of colored boxes; each individual from two groups is represented by a single column. Red color indicates up-regulated while green indicates down-regulated. miRNAs that were expressed at higher levels are assigned progressively brighter shades of red while miRNAs expressed at lower levels are assigned progressively brighter shades of green.

Warner-Bratzler shear forces (WBSF) measurements were made to evaluate variation of beef tenderness caused by acute stress. The results showed that the average WBSF for stressed group and control group were 19.74 kg and 5.04 kg, respectively. The stressed group was much tougher than the control (non-stressed) group from the student *t*-test result (*P* ≪ 0.0001). To determine the miRNA expression patterns in Angus cattle with different stress status, miRNA microarray analysis was conducted on LD muscle. These arrays were designed based on the miRBase Version 11.0 and contained 126 bovine miRNAs. For each miRNA, there were 4 to 8 repeat probes on each slide. After hybridization, washing, scanning, data were collected and then Limma package was applied. A total of 13 miRNAs were identified as differentially expressed miRNAs using the criteria of *P* value less than 0.05 and FDR (false discover rate) less than 0.4 (Table 1). Of these, one miRNA was down-regulated while 12 miRNAs were up-regulated in stressed group compared with control group. To reveal the overall expression profiles of these differentially expressed miRNAs in these two groups, clustering analysis was performed as previously described. The visualization showed that the expression patterns of these miRNAs can apparently divide these 6 individuals into stressed and control groups (Figure 1). To obtain a highly statistically confident result, a stringent statistical significance threshold (*P* ≪ 0.05, Fold Change ≫ 1.5) was used. Bta-miR-497 was chosen as the most significantly differentially expressed miRNA to do further analysis.

**Table 1:** Differentially expressed miRNAs between stressed group and control in Angus cattle

Name	Fold Change	*P* Value	adj.*P* Value
bta-miR-151	0.839	0.012	0.397
bta-miR-93	1.128	0.023	0.397
bta-miR-10b	1.183	0.037	0.397
bta-miR-20b	1.187	0.035	0.397
bta-let-7c	1.200	0.039	0.397
bta-let-7a	1.206	0.015	0.397
bta-miR-181b	1.215	0.043	0.397
bta-miR-99a	1.226	0.009	0.397
bta-miR-195	1.244	0.046	0.397
bta-miR-19b	1.248	0.027	0.397
bta-miR-660	1.292	0.020	0.397
bta-miR-125b	1.321	0.004	0.397
bta-miR-497	1.624	0.023	0.397

Fold change was computed as (stressed/control).

### qPCR analysis of differentially expressed miRNA

**Figure 2:**
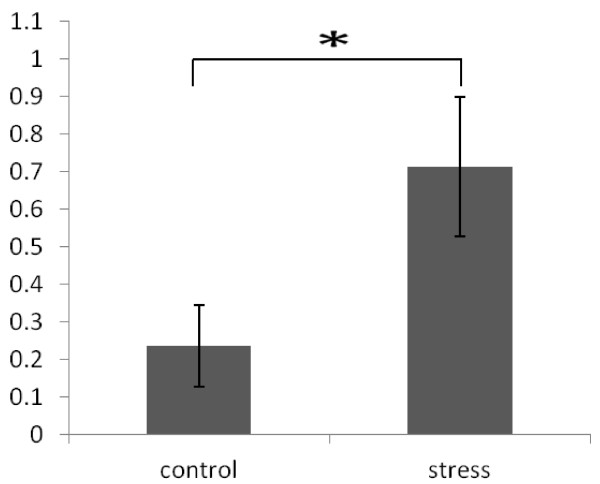
**Expression of*bta-miR-497*in control and stressed groups was measured by qRT-PCR and normalized to*U6*.** The quantitative results are represented as mean ± SEM (n = 3). A single asterisk (*P* ≪0.05).

To validate the microarray results, mimic bta-miR-497 were synthesized by miScript Primer Assays. Quantitative RT-PCR was performed to measure the expression level of bta-miR-497 in stressed and control groups. The results showed that the expression of bta-miR-497 significantly increased in stressed group compared to control group (*P* ≪ 0.05) (Figure 2), consistent with the miRNA microarray results (fold change = 1.62 in microarray), namely, the expression of bta-miR-497 was increased after the acute stress.

### Prediction of targets of differentially expressed miRNA and function annotation

To understand the potential functions of significantly differentially expressed miRNA in this diverse stress status, 135 genes were predicted as the potential targets of miR-497 in bovine by using bioinformatics method. To further explore the function of these predicted target genes, Gene Ontology analysis was performed. The results showed that the predicted target genes in GO biological process terms were enriched in cellular catabolic process and cellular process. In cellular component category, GO terms related to the cytoplasmic part, membrane-bounded organelle, intracellular membrane-bounded organelle, organelle, intracellular organelle, cytoplasm and intracellular organelle part. The molecular function category of GO terms showed that succinyltransferase activity, purine nucleotide binding, ribonucleotide binding, purine ribonucleotide binding, purine ribonucleoside triphosphate binding, GTP binding, guanyl nucleotide binding, guanyl ribonucleotide binding, S-acyltransferase activity and GTPase activity were enriched. Summaries of the enriched GO term categories for predicted target genes are shown in the Table 2.

**Table 2:** Significant GO terms predicted target genes were involved in

GOID	Ontology	Term	q	p
GO:0044248	biological_process	cellular catabolic process	13	0.009486
GO:0009987	biological_process	cellular process	65	0.043591
GO:0044444	cellular_component	cytoplasmic part	38	0.008829
GO:0043227	cellular_component	membrane-bounded organelle	48	0.03211
GO:0043231	cellular_component	intracellular membrane- bounded organelle	48	0.03211
GO:0043226	cellular_component	Organelle	53	0.035303
GO:0043229	cellular_component	intracellular organelle	53	0.035303
GO:0005737	cellular_component	Cytoplasm	45	0.041648
GO:0044446	cellular_component	intracellular organelle part	32	0.044642
GO:0016748	molecular_function	succinyltransferase activity	3	0.00289
GO:0017076	molecular_function	purine nucleotide binding	25	0.009486
GO:0032553	molecular_function	ribonucleotide binding	25	0.009486
GO:0032555	molecular_function	purine ribonucleotide binding	25	0.009486
GO:0035639	molecular_function	purine ribonucleoside triphosphate binding	25	0.009486
GO:0005525	molecular_function	GTP binding	11	0.009486
GO:0019001	molecular_function	guanyl nucleotide binding	11	0.009876
GO:0032561	molecular_function	guanyl ribonucleotide binding	11	0.009876
GO:0016417	molecular_function	S-acyltransferase activity	3	0.013341
GO:0003924	molecular_function	GTPase activity	7	0.03211

**Figure 3:**
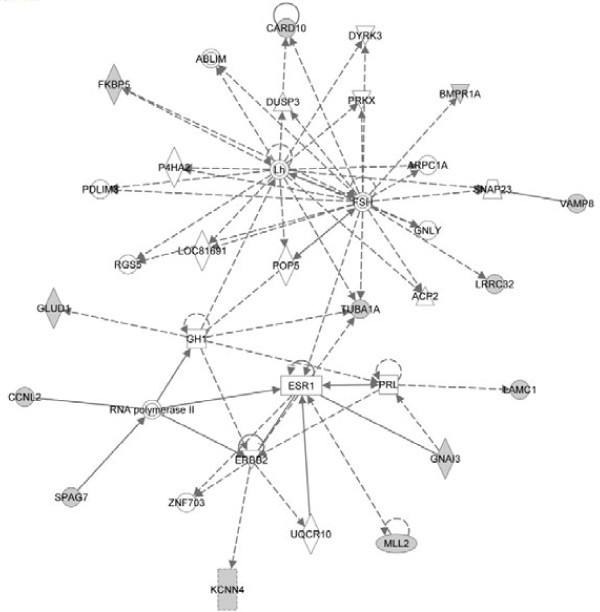
**The top 1# network target genes involved.** Solid line represents direct interaction and dash line represents indirect interaction.

**Figure 4:**
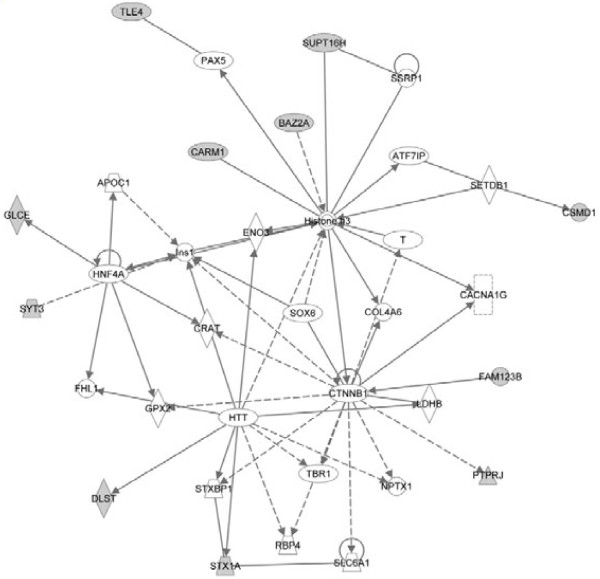
**The top 2# network target genes involved.** Solid line represents direct interaction and dash line represents indirect interaction.

**Figure 5:**
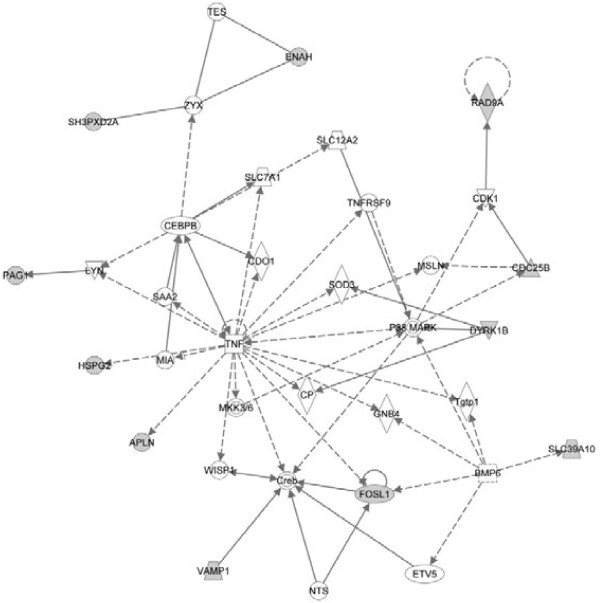
**The top 3# network target genes involved.** Solid line represents direct interaction and dash line represents indirect interaction.

To further visualize the pathways and networks these target genes related with, IPA of target genes was conducted. Analysis results showed that cell cycle, cell morphology, cellular function and maintenance, molecular transport and cellular movement were ranked in the top of “Molecular and Cellular Functions”. While, inhibition of angiogenesis by TSP1, D-glutamine and D-glutamate metabolism, G2/M DNA damage checkpoint regulation, galactose metabolism and nucleotide sugars metabolism were among the top canonical pathways. The most significant networks functioned in drug metabolism, endocrine system development and function, lipid metabolism, amino acid metabolism, molecular transport, small molecule biochemistry, gene expression, cellular movement, cell cycle, cardiovascular system development and function, organismal development, cancer and gastrointestinal disease. Summaries of the enriched networks and their functions are shown in Table 3 and graphical networks are represented (Figure 3, 4 and 5).

**Table 3:** Networks and functions that target genes are related with

ID	Score	Focus Molecules	Top Functions
1	19	13	Drug Metabolism, Endocrine System Development and Function, Lipid Metabolism
2	15	11	Amino Acid Metabolism, Molecular Transport, Small Molecule Biochemistry
3	15	11	Gene Expression, Cellular Movement, Cell Cycle
4	15	11	Cardiovascular System Development and Function, Organismal Development, Cancer
5	15	11	Cell Cycle, Cancer, Gastrointestinal Disease
6	11	9	Cardiovascular Disease, Gene Expression, Hematological Disease
7	2	1	Carbohydrate Metabolism, Lipid Metabolism, Small Molecule Biochemistry
8	2	1	Cellular Growth and Proliferation, Gene Expression, Infectious Disease
9	2	1	RNA Post-Transcriptional Modification, Cellular Compromise, Cellular Development
10	2	1	Cancer, Embryonic Development, Neurological Disease
11	2	1	RNA Damage and Repair, Nutritional Disease, Organismal Injury and Abnormalities
12	2	1	Cellular Growth and Proliferation, Developmental Disorder, Embryonic Development
13	2	1	Developmental Disorder, Genetic Disorder, Metabolic Disease
14	2	1	Cell Signaling, Cellular Assembly and Organization, Cellular Function and Maintenance
15	2	1	Post-Translational Modification, Protein Synthesis, Cell-To-Cell Signaling and Interaction
16	2	1	Cellular Assembly and Organization, Cellular Function and Maintenance, Cellular Movement
17	2	1	Cellular Assembly and Organization, Cellular Function and Maintenance, Cell-To-Cell Signaling and Interaction
18	2	1	Cell Cycle, Cellular Movement, Embryonic Development
19	2	1	Infectious Disease, DNA Replication, Recombination, and Repair, Gene Expression

ID represents the network No. Score means gene number in this network and Focus molecular means the target gene number in this network.

## Discussion

Abnormal or disease conditions can induce dysregulation of mRNA and protein levels. It has been reported that muscle-specific miRNAs, miR-206 and miR-499, are upregulated and miR-1, miR-133a, and miR-133b are downregulated in extraocular muscles compared to limb muscle, concluding that a miRNA network contributes to the extraocular muscles by regulating posttranscriptional expression of genes involved in structure, signaling, metabolism, angiogenesis, myogenesis, and regeneration in extraocular muscles [7]. In addition, miR-145 is found to be necessary for myocardin-induced reprogramming of adult fibroblasts into smooth muscle cells and can induce differentiation of multipotent neural crest stem cells into vascular smooth muscle [10]. Meanwhile, miR-145 and miR-143 cooperatively target a network of transcription factors to promote differentiation and repress proliferation of smooth muscle cells [10]. Both also act as integral components of the regulatory network whereby serum response factor controls cytoskeletal remodeling and phenotypic switching of smooth muscle cells during vascular disease [29]. In our study, several miRNAs were found to be dysregulated due to different stress status, of which, some have been previously studied. For example, miR-497 has been found to promote ischemic neuronal death by negatively regulating antiapoptotic proteins [30]. Another research found that miR-497 and miR-302b co-regulate ethanol-induced neuronal cell death through BCL2 protein and cyclin D2 [31]. But its function in muscle development has not been reported yet. Therefore, these finding further suggest that miRNAs may play some roles on transcriptional circuits controlling gene expression in skeletal muscle.

Notably, the surgical implantation of rumen canulas imitated a non fatal form of hardware disease. Hardware disease occurs when an animal ingests a sharp piece of metal and the metal pierces the rumen or reticulum wall. As expected, the phenotype in this study indicated that those animals undergoing this stress had significantly higher WBSF. In this research, we identified differently expressed miRNAs associated with divergent stress status in LD muscles samples between stressed and control groups. The annotation of predicted target genes further showed that miRNA may be involved in important pathways regulating target genes, such as lipid metabolism, amino acid metabolism, gene expression, molecular transport, *etc*. In the future, the predicted miRNA targets need to be validated *in vitro* and the expression levels of corresponding target genes and proteins should be measured, which will help to elucidate how miRNAs regulate gene transcription and protein expression in the variation of beef quality and tenderness.

## Materials and methods

### Sample collection and experiment design

Seven purebred Angus steers were obtained from Wye Angus farm (Queenstown, MD). After weaning the steers were acclimated to a pelleted forage diet only to meet maintenance needs. At 10 months of age, 4 steers underwent a surgical procedure that involved anesthetization and placement of a rumen catheter. The surgery was acute stress compared to normal growth condition. Three steers that received no surgery were designated as control group. At the age of 1 year, the steers were harvested. After harvest 10 mg *longissimus dorsi* (LD) muscle from the 12^th^ to 13^th^ rib of the right side of the carcass were placed in RNAlater solution (Qiagen, Valencia, CA) and stored at −80°C for further analysis. Steaks of the LD from the 12^th^ to 13^th^ rib of the left side of the carcass were obtained, vacuum packed, stored at 4°C for a total of 14 days post harvest, and then frozen at −20°C. Once all steaks were obtained, aged, the steaks were thawed at 4°C, cooked to an internal temperature at 70°C, cooled, cored and then analyzed for WBSF as previously described [32]. After WBSF data were analyzed by student *t*-test, three extremely tough individuals were chosen to be designed as stressed group and three cattle without stress were designed as control group. Based on these tough and control groups, a total of 6 miRNA microarrays were hybridized and analyzed. All procedures were approved by the University of Maryland Institutional Animal Care and Use Committee (Protocol # R-07-05).

### RNA extraction and miRNA microarray hybridization

Total RNAs from the 6 samples were extracted using miRNAeasy Mini Kit (Qiagen) as described in the manufacturer’s instructions. The RNAs were quantified by NanoDrop ND 1000 Spectrophotometer (Thermo-scientific, Wilmington, DE) and RNA integrity determined by 2100 Bioanalyzer (Agilent Technologies, Santa Clara, CA). Equal aliquots of total RNA from each sample were pooled together as common reference RNA. One μg total RNA from each sample or common reference were labeled with Hy3^TM^ and Hy5^TM^ fluorescent label, respectively, with the help of the miRCURY^TM^ LAN Array power labeling kit (Exiqon, Denmark) following the instructions. The Hy3^TM^-labeled samples and a Hy5^TM^-labeled reference RNA sample were mixed pair-wise and hybridized to the miRCURY^TM^ LNA array (Version 9.2; Exiqon, Denmark), which contained capture probes targeting all of the miRNAs for all the species registered in the miRBase (Version 11.0) at the Sanger Institute. One hundred and twenty-six of these probes are bovine-related miRNAs in the miRBase version. Hybridization was performed according to the miRCURY^TM^ LNA array manual with a Tecan HS4800 hybridization station (Tecan, Austria). After hybridization, the microarray slides were scanned and stored in an ozone free environment to prevent potential bleaching of the fluorescent dyes. The miRCURY^TM^ LNA array microarray slides were scanned using the Agilent G2565BA Microarray Scanner System (Agilent) and image analysis was performed with ImaGene 8.0 software (BioDiscovery, Inc., USA).

### miRNA microarray data analysis

Microarray data were analyzed in R using the Linear models for microarray data (Limma) package. For each miRNA, quantified signals within arrays were averaged. Normalizations within arrays and between arrays were performed using the global LOWESS (LOcally WEighted Scatterplot Smoothing) regression algorithm. Contrasts were made to compare stressed and control groups. Differentially expressed miRNAs were selected to do further analysis using the stringent statistic criteria of *p* value less than 0.05 and FDR (false discover rate) less than 0.4.

### qRT-PCR analysis of miRNA expression

Total mRNAs including miRNAs were extracted from 6 same samples using miRNeasy Mini Kit (QIAGEN) and RNeasy Mini Kit (QIAGEN) according to the standard protocol. mRNAs were reversely transcribed and quantified with miScript Reverse Transcription Kit (QIAGEN), miScript SYBR Green PCR Kit (QIAGEN), and miScript Primer assays (QIAGEN). In the reverse transcription control, PCR water (Invitrogen) was used to replace miRNA samples. Briefly, 1μg of purified miRNA was used for reverse transcription, and then diluted to 5 volumes. Two μl of diluted RT products were used for real-time PCR quantification. Two types of controls were applied in real-time PCR, including reverse transcription control and blank using PCR water, to ensure that no amplicon was observed in the controls. U6 were used as normalization controls. Data were analyzed using the 2^-ΔΔCT^ method and student T tests were used to compare the miRNAs expression levels (SAS version 9.2).

Here we only validated the most significant miRNA, namely bta-miR-497, which sequence is shown as CAGCAGCACACUGUGGUUUGUA. The mimic miRNA of bta-miR-497 was synthesized by Qiagen.

### Prediction of miRNA targets

The target genes for miRNAs were predicted by TargetScanHuman (http://www.targetscan.org/vert_50/). In the menu of “Select a species”, cow was chosen and the names of significantly differentially expressed miRNAs were inputted and then submitted. From the output only the genes with the conserved sites were reserved as predicted target genes of this miRNA.

### Data mining and network analysis of significantly differentially expressed miRNAs and predicted target genes

Hierarchical clustering of significantly differentially expressed miRNAs was performed using Cluster 3.0 [33]. The expression data were further filtered, adjusted and normalized. Average linkage clustering was performed and visualized using Treeview. The initial information on Gene Ontology [15] functions and functional relevance of predicted target genes was obtained from Gene Ontology Enrichment Analysis Software Toolkit (GOEAST) [34]. The GO analysis included biological process, molecular function and cellular component. Ingenuity Pathway Analysis (IPA, Ingenuity System, Redwood City, CA) was used to generate networks and assess statistically relevant biofunctions and canonical pathways that predicted target genes are involved in. These genes were mapped to corresponding genes in the Ingenuity knowledge database. The biofunctional analysis identified the molecular and cellular function, physiological system development and function. Canonical Pathway Analysis identified the most significant pathways in the dataset.

## Competing interest

The authors have declared that no competing interest exist.

## Authors’ contributions

ZCP, TF extracted RNA, performed array hybridization and partial data analysis. ZCP analyzed the microarray data and wrote manuscript. SU performed the WBSF. JZS and SU designed the experiments and revised the paper. All authors read and approved the final manuscript.
